# A low cost and high performance polymer donor material for polymer solar cells

**DOI:** 10.1038/s41467-018-03207-x

**Published:** 2018-02-21

**Authors:** Chenkai Sun, Fei Pan, Haijun Bin, Jianqi Zhang, Lingwei Xue, Beibei Qiu, Zhixiang Wei, Zhi-Guo Zhang, Yongfang Li

**Affiliations:** 1 CAS Research/Education Center for Excellence in Molecular Sciences, CAS Key Laboratory of Organic Solids, Institute of Chemistry, Chinese Academy of Sciences, 100190 Beijing, China; 2 School of Chemical Science, University of Chinese Academy of Sciences, 100049 Beijing, China; 3 CAS Key Laboratory of Nanosystem and Hierarchical Fabrication, CAS Center for Excellence in Nanoscience, National Center for Nanoscience and Technology, 100190 Beijing, China; 4 Laboratory of Advanced Optoelectronic Materials, College of Chemistry, Chemical Engineering and Materials Science, Soochow University, Suzhou, 215123 Jiangsu China

## Abstract

The application of polymer solar cells requires the realization of high efficiency, high stability, and low cost devices. Here we demonstrate a low-cost polymer donor poly[(thiophene)-alt-(6,7-difluoro-2-(2-hexyldecyloxy)quinoxaline)] (PTQ10), which is synthesized with high overall yield of 87.4% via only two-step reactions from cheap raw materials. More importantly, an impressive efficiency of 12.70% is obtained for the devices with PTQ10 as donor, and the efficiency of the inverted structured PTQ10-based device also reaches 12.13% (certificated to be 12.0%). Furthermore, the as-cast devices also demonstrate a high efficiency of 10.41% and the devices exhibit insensitivity of active layer thickness from 100 nm to 300 nm, which is conductive to the large area fabrication of the devices. In considering the advantages of low cost and high efficiency with thickness insensitivity, we believe that PTQ10 will be a promising polymer donor for commercial application of polymer solar cells.

## Abstract (web summary)

A problem that hinders the commercialization of polymer solar cells is the complication in synthesis and thus the low yield and high cost of the polymers. Here, Sun et al. synthesize a new polymer via a two-step process with a yield close to 90% and show high photovoltaic performance with efficiency of 12%.

## Introduction

Polymer solar cells (PSCs) have received widespread interests and have developed quickly in recent years because of its advantages of solution processing, light weight and flexibility in comparison with the traditional silicon-based solar cells^1,2^. Active layer of the PSCs is composed of a *p*-type conjugated polymer as donor blending with a fullerene derivative or a nonfullerene *n*-type organic semiconductor (*n*-OS) as acceptor^3–5^. Photovoltaic power conversion efficiency (PCE), stability, and cost are the three most crucial issues that must be taken into account for the commercial application of PSCs. And the donor and acceptor photovoltaic materials play essential role for increasing PCE, improving stability and decreasing cost of the PSCs.

The concept of bulk-heterojunction PSCs was first proposed in 1995 (ref. ^3^), and PCE of the PSCs at that time was only ca. 1% which is far from considering application. Since then, therefore, the researchers mainly focused on increasing PCE of the PSCs by developing efficient photovoltaic materials (donor^6–8^ and acceptor^9,10^) and efficient electrode buffer layer materials^11–16^, and optimizing active layer morphology^17–19^, etc. For the PSCs with fullerene derivatives (especially PC_71_BM) as acceptors, PCE has gradually increased to over 10% recently by designing low-bandgap polymer donors such as PTB7-Th^20^, PNTz4T^21^, and PffBT4T-C_9_C_13_ (ref. ^22^), etc. In recent 2 years, low-bandgap nonfullerene *n*-OS acceptors, such as IDTBR^23^, *m*-ITIC^24^, IDIC^25^, etc., have attracted great attention owing to their advantages of broad and strong absorption, easy tuning electronic energy levels, and high morphology stability in comparison with the fullerene derivative acceptors^26^. PCE of the PSCs with *n*-OS as acceptors has rapidly increased to over 11% by using medium bandgap polymer donors such as J71 (ref. ^27^), PBDB-T^28^, PB3T^29^, etc.

Now, the PCE has reached the threshold for application. Next step, we should consider the stability and cost issues for commercial application of the PSCs. However, very few efforts have been made on reducing the costs of the photovoltaic materials, and costs of the efficient polymer donors reported so far were too high to meet commercial application of the PSCs due to their complicated molecular structures, verbose multi-steps synthesis, and multiple purifications^30^. Actually, poly(3-hexylthiophene) (P3HT) is still the main donor material for the fabrication of large area PSCs^31^, because it can be synthesized in large scale with relatively low cost. However, the photovoltaic performance of P3HT is poor^32,33^. Hence, developing low-cost and efficient polymer donors becomes one of the greatest challenges for the application of PSCs.

**Fig. 1:**
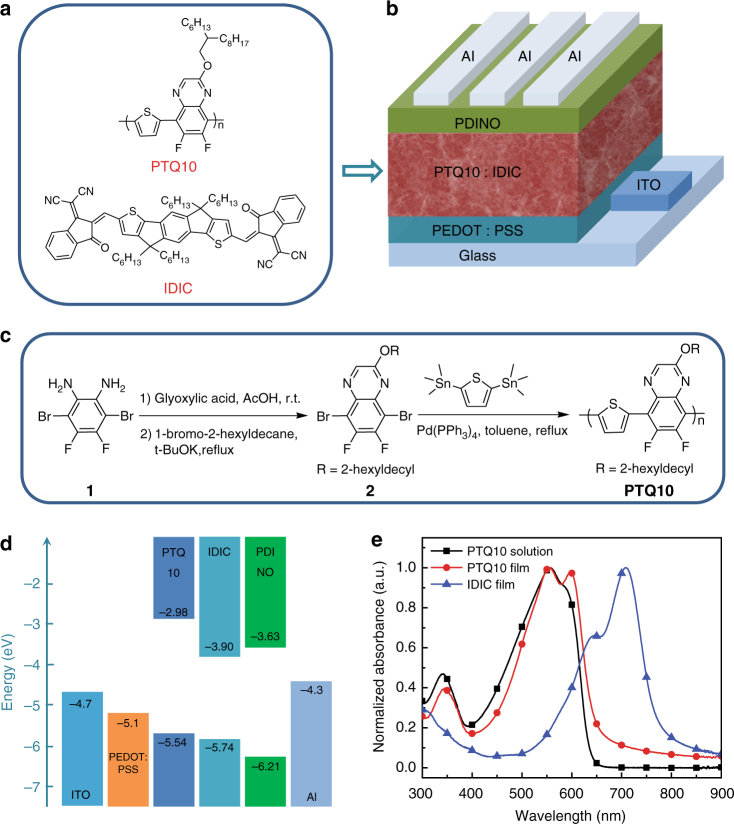
Photovoltaic materials and device structure of the PSCs. **a** Molecular structures of the polymer donor PTQ10 and the *n*-OS acceptor IDIC. **b** Devices architecture of the traditional structured PSCs. **c** Synthetic route of PTQ10. **d** Energy level diagram of the related materials used in the PSCs. **e** Normalized absorption spectra of the donor PTQ10 and the acceptor IDIC

Herein, we design and synthesize a low-cost polymer donor poly [(thiophene)-alt-(6,7-difluoro-2-(2-hexyldecyloxy)quinoxaline)] (PTQ10) (Fig. 1a). The molecular design strategy of PTQ10 is based on the donor–acceptor (D–A) copolymerization concept, using simple thiophene ring as donor unit and difluorine-substituted quinoxaline as acceptor unit. The alkoxy side chain on quinoxaline unit is to ensure good solubility and to enhance absorption of the polymer, while the difluorine substituents are for down-shifting the highest occupied molecular orbital (HOMO) energy level and increasing hole mobility of the polymer donor^34^. PTQ10 possesses a simple molecular structure and can be synthesized with low cost and high overall yield of 87.4% via only two-step reactions from cheap raw materials. More importantly, the optimized PSCs with PTQ10 as donor and an *n*-OS IDIC as acceptor demonstrate an impressive PCE of 12.70% which is one of the highest PCE among the single-junction PSCs reported so far, and the as-cast devices without any post-processing also demonstrate a high PCE of 10.41%. Furthermore, the devices have good reproducibility and have high tolerance of the active layer thickness with a PCE over 10% even at an active layer thickness of 310 nm. The results indicate that PTQ10 is a promising polymer donor for commercial products, and it will make the application of PSCs highly promising.

## Results

### Synthesis and characterization of PTQ10

The synthetic route of PTQ10 is depicted in Fig. 1c, and the detailed synthesis procedures are described in the Methods section. The synthetic route of monomer 2 was carefully designed with cheap raw material and efficient reaction for realizing low-cost synthesis. PTQ10 possesses good solubility in common organic solvents. Its thermal decomposition temperature (*T*_d_) at 5% weight loss is measured to be 383 °C (Supplementary Fig. 1a), indicating its good thermal stability for the application in PSCs.

Electronic energy levels of PTQ10 were measured by electrochemical cyclic voltammetry. The *E*_HOMO_ and *E*_LUMO_ of PTQ10 were calculated to be −5.54 eV and −2.98 eV (Fig. 1d) from onset oxidation and onset reduction potentials, respectively (Supplementary Fig. 1b). Figure 1e shows UV–vis absorption spectra of PTQ10 in chloroform solution and in thin film, and the absorption spectrum of IDIC film for comparison. PTQ10 film displays a strong absorption from 450 to 620 nm with an absorption edge at 645 nm which corresponds to an optical bandgap of 1.92 eV. PTQ10 and IDIC films display complementary absorption in the wavelength region from 400 to 800 nm, which will benefit to the solar light harvest for the PSCs with PTQ10 as donor and IDIC as acceptor.

### Photovoltaic properties

**Fig. 2:**
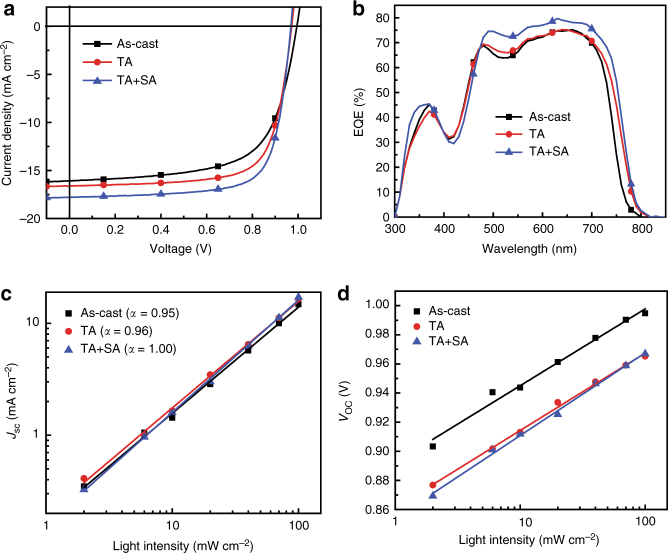
Photovoltaic performance of the PSCs based on PTQ10: IDIC. **a** *J*–*V* curves of the traditional structured PSCs based on PTQ10: IDIC (1:1, w/w), under the illumination of AM1.5G, 100 mW cm^−2^. **b** EQE spectra of the corresponding PSCs. The dependence of *J*_sc_ (**c**) and *V*_oc_ (**d**) on light intensity (*P*_light_) of the optimized PSCs

In order to investigate photovoltaic properties of PTQ10, we fabricated the traditional structured PSCs with PTQ10 as donor, *n*-OS IDIC as acceptor, PEDOT: PSS (poly(3,4-ethylenedioxythiophene):poly(styrene-sulfonate) as anode buffer layer and PDINO (perylene diimide functionalized with amino N-oxide) as cathode buffer layer^16^ (Fig. 1b). It should be mentioned that IDIC^25,35^ was selected as acceptor because it possesses a simpler structure with alkyl side chains on its smaller fused ring core and relatively low-cost synthesis in comparison with the widely used *n*-OS acceptors ITIC^10^, etc. Photovoltaic performances of the PSCs were optimized by using different donor/acceptor weight ratio and different active layer thickness, and by the treatment of thermal annealing (TA) and solvent vapor annealing (SA). The optimized device fabrication conditions include the donor/acceptor weight ratio of 1:1, active layer thickness of 130 nm, TA at 140 °C for 5 min, and SA by chloroform solvent for 30 s. Figure 2a shows current density–voltage (*J*–*V*) curves of the optimal PSCs based on PTQ10: IDIC with or without TA and SA treatments, and the corresponding photovoltaic parameters are listed in Table 1.

**Table 1:** Photovoltaic parameters of the PSCs based on PTQ10: IDIC

Devices	*V*_oc_ (V)	*J*_sc_ (mA cm^−2^)	FF (%)	PCE (%)
As-cast^a^	0.995 (0.995 ± 0.003)^d^	16.07 (15.70 ± 0.16)	65.10 (64.89 ± 0.45)	10.41 (10.14 ± 0.14)
TA^a^	0.972 (0.962 ± 0.004)	16.61 (16.61 ± 0.22)	72.13 (71.55 ± 0.71)	11.65 (11.43 ± 0.10)
TA + SA^a^	0.969 (0.962 ± 0.005)	17.81 (17.44 ± 0.30)	73.60 (73.26 ± 0.63)	12.70 (12.29 ± 0.18)
TA + SA^b^	0.960 (0.960 ± 0.003)	19.65 (19.69 ± 0.27)	64.29 (63.55 ± 1.30)	12.13 (12.01 ± 0.10)
TA + SA^c^	0.96	20.12	62.1	12.0

^a^Traditional structured PSCs with donor:acceptor weight ratio of 1:1

^b^Inverted structured PSCs with a device structure of ITO /ZnO /PTQ10: IDIC /MoO_3_ /Ag and with donor:acceptor weight ratio of 1:1.5

^c^Confirmed photovoltaic performance of the inverted PSCs by NIM

^d^Data in parentheses are average values calculated from more than 20 devices

It can be seen from Table 1 that all of the PSCs exhibit high *V*_oc_ of 0.960~0.995 V, which should be benefitted from the lower-lying *E*_HOMO_ (−5.54 eV) of PTQ10. The *V*_oc_ decreased from 0.995 V to 0.972 V and 0.969 V when the devices were treated with TA and TA + SA, respectively, which could be ascribed to the red-shifted absorption (the reduced optical band gap) of PTQ10 and IDIC treated with TA and TA + SA (Supplementary Fig. 2a and 2b). The as-cast PSCs without post-processing show an impressive PCE of 10.41%, and the PCE increased to 11.65% and 12.70%, respectively, after TA and TA + SA treatments. To our knowledge, the PCE of 12.70% is one of the highest efficiencies among the single-junction PSCs reported to date. In addition, it is worth noticing that PCE of 10.41% for the as-cast devices is the highest efficiency in the PSCs without post-treatments, and the simple device fabrication process for the as-cast devices will significantly reduce device fabrication costs, which is very important for future industrial production of the PSCs.

The external quantum efficiency (EQE) spectra of the optimized devices with different post-processing treatments are shown in Fig. 2b. All the three PSCs exhibit broad light response with high EQE values over 50% from 450 to 730 nm, which means high photoelectric conversion efficiency in the PTQ10: IDIC blend films. The current density values integrated from the EQE spectra under the AM 1.5G spectrum are 15.30 mA cm^−2^ for the as-cast device, 16.01 mA cm^−2^ for the TA-treated device, and 17.08 mA cm^−2^ for the TA + SA-treated device, which are consistent quite well with the *J*_sc_ values obtained from *J*–*V* curves within 4% mismatch, indicating the reliability of the measured *J*_sc_ data. The enhanced EQE value and current density of the PSCs with the TA or TA + SA treatments should be ascribed to its red-shifted absorption, enhanced absorption coefficient (Supplementary Fig. 2c), and the broadened EQE spectra (Fig. 2b) in comparison with that of the blend films without the treatment (as-cast).

In order to confirm the high PCE of the PTQ10-based PSCs, we fabricated the inverted structured PSCs with the device structure of ITO/ZnO/PTQ10: IDIC/MoO_3_/Ag, in considering the better stability of the inverted PSCs for sending the devices out to the National Institute of Metrology (NIM) of China for the efficiency confirmation. The inverted PSC based on PTQ10: IDIC (1:1.5, w/w) with active layer thickness of 130 nm and with the treatment of TA at 140 °C for 5 min and SA by chloroform solvent for 30 s showed a PCE of 12.13% with a *V*_oc_ of 0.960 V, a *J*_sc_ of 19.65 mA cm^−2^, and an fill factor (FF) of 64.29%, as shown in Table 1 and in Supplementary Fig. 3. The PCE of the inverted PSCs was confirmed to be 12.0% by NIM (see the Test Report of NIM in Supplementary Fig. 4 and the last line in Table 1). The slightly lower PCE of the inverted device is due to its lower fill factor which could be ascribed to the un-optimized cathode and anode buffer layer materials of the inverted PSCs.

Batch to batch variation of the polymers is an unfavorable factor to the commercial application of PSCs^36^. Supplementary Table 1 lists the photovoltaic parameters of the optimized traditional structured PSCs based on PTQ10: IDIC with using the PTQ10 samples synthesized in five batches to investigate the photovoltaic repeatability of PTQ10. The photovoltaic performance of PTQ10 show less batch to batch variation with the PCE values ranging from 11.90% to 12.70%, which indicates the good photovoltaic repeatability of PTQ10.

The hole mobility (*μ*_h_) and electron mobility (*μ*_e_) of the PTQ10: IDIC blend layers without (as-cast) and with the TA and TA + SA treatments were measured using space charge limited current (SCLC) method with hole-only (ITO/PEDOT: PSS /PTQ10: IDIC/Au) and electron-only (ITO/ZnO/PTQ10: IDIC/PDINO/Al) devices, and the measurement results are shown in Supplementary Fig. 5. For the as-cast PTQ10: IDIC blend films, *μ*_h_ and *μ*_e_ are 0.36 × 10^–4^ cm^2^ V^−1^ s^−1^ and 3.43 × 10^–4^ cm^2^ V^−1^ s^−1^, respectively, with *μ*_e_/*μ*_h_ of 9.53. While *μ*_h_ and *μ*_e_ increased to 3.21 × 10^–4^ cm^2^ V^−1^ s^−1^ and 4.80 × 10^–4^ cm^2^ V^−1^ s^−1^, respectively, after TA treatment with a balanced *μ*_e_/*μ*_h_ of 1.50. With TA + SA treatment, the *μ*_h_ and *μ*_e_ values were further improved to 5.04 × 10^–4^ cm^2^ V^−1^ s^−1^ and 6.72 × 10^–4^ cm^2^ V^−1^ s^−1^, respectively, with more balanced *μ*_e_/*μ*_h_ ratio of 1.33. The increased and more balanced charge mobility suggests better charge transfer capability of the PTQ10: IDIC blend films after TA or TA + SA treatments. This finding together with the enhanced absorption and the reduced charge carrier recombination after TA + SA treatment could be collectively responsible for the improved *J*_sc_ and FF of the optimized devices.

For application of the PSCs, stability of the devices is one of the crucial issues besides photovoltaic performance and cost^37^. Here, the device stability was tested for the inverted PSCs based on PTQ10: IDIC with simple encapsulation by ultraviolet-curable epoxy and thin glass slides. Supplementary Fig. 6 shows the results of devices stability experiments. The PCE of the inverted-structured PSCs based on PTQ10: IDIC remained 88.27% and 87.82% of their initial value after approximately 1000 h of storage under N_2_ and air atmosphere, respectively. Then the efficiency remain almost unchanged in the following 1000 h. The results indicate good stability of the PSCs based on PTQ10: IDIC.

### Charge carrier recombination

To understand the effect of TA or TA + SA treatments on the enhanced photovoltaic performance, the charge carrier recombination behavior in the traditional structured PSCs was studied by measuring the dependence of *J*_sc_ and *V*_oc_ on light intensity (*P*_light_). The relationship of *J*_sc_ and *P*_light_ can be depicted by the formula *J*_sc_ ∝(*P*_light_)^*α*^, where the value of *α* indicates the degree of bimolecular recombination. The value of *α* should be 1 when bimolecular recombination do not occur in donor/acceptor blend films, and there is some bimolecular recombination if *α* value is smaller than 1 (ref. ^38^). Figure 2c displays the plots of log *J*_sc_ versus log *P*_light_, and *α* values are 0.95, 0.96, and 1.00 for the devices without (as-cast), with TA, and with TA + SA treatments, respectively. The gradually increased values of *α* indicate the reduced bimolecular recombination when the blend films are processed with TA and TA + SA compared to the as-cast devices. Especially, *α* value of 1 for the PSCs with TA + SA treatment indicates that there is no bimolecular recombination in the TA + SA treated devices. For the as-cast devices and TA-treated devices, another plausible reason for the deviation of the *α* values from unity, can be understood in term of the build-up of space-charge in the device due to the unbalanced electron-hole mobility as indicated by Blom’s work^39,40^. Figure 2d shows the plots of *V*_oc_ versus ln (*P*_light_) of the PSCs. If bimolecular recombination is the exclusive recombination form, the slope of the fitting straight line of *V*_oc_ versus ln (*P*_light_) should be *kT*/*e* (where *e* is the elementary charge, *k* is the Boltzmann constant, and *T* is the Kelvin temperature)^41^. The slopes of the fitting lines for the as-cast, TA-treated, and TA + SA-treated devices are 0.920*kT/e*, 0.924*kT/e*, and 0.988*kT/e*, respectively. The slope very close to *kT/e* for the TA + SA-treated PSCs indicates that almost no other recombination occurs in the devices with TA + SA treatment. The results of *J*_sc_ and *V*_oc_ dependence on *P*_light_ indicate that there are very little charge carrier recombination in the optimized PSCs treated with TA + SA, which consequently results in the best PCE of 12.70% for the PSCs with TA + SA treatment.

### Morphological characterization

**Fig. 3:**
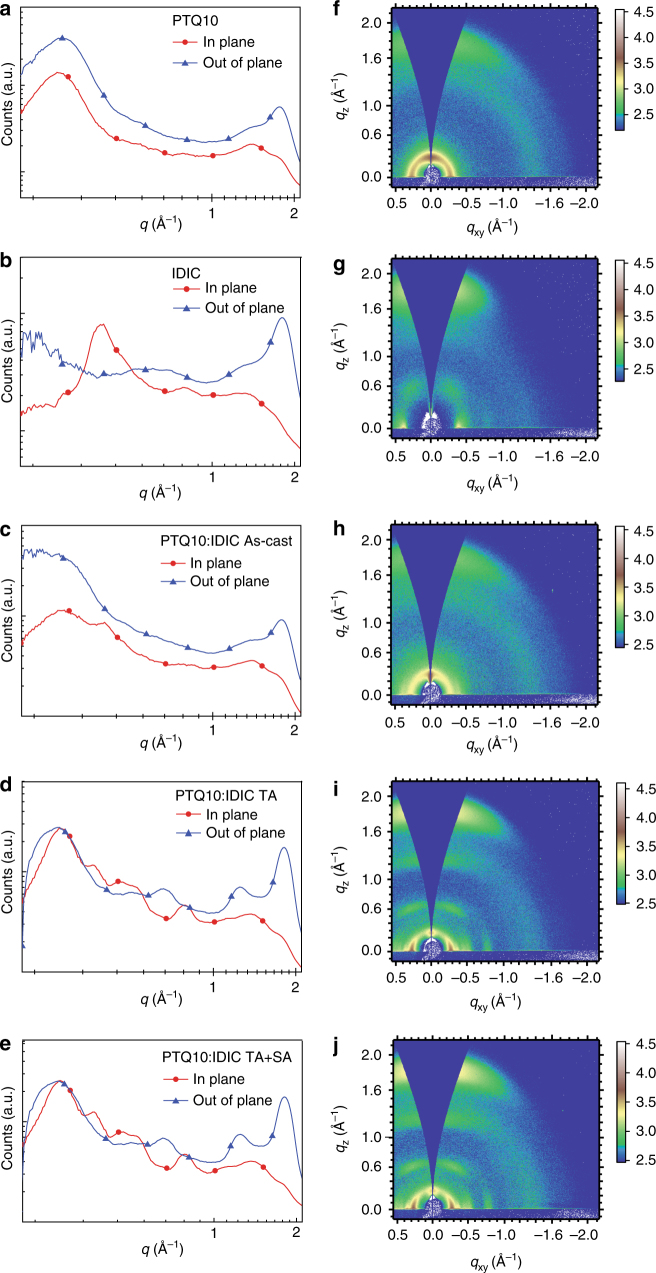
Plots and images of the GIWAXS measurements. Line cuts of the GIWAXS images of neat PTQ10 film (**a**), neat IDIC film (**b**), and PTQ10: IDIC blend films without (as-cast) (**c**), with TA treatment (**d**), and with TA + SA treatment (**e**). GIWAXS images of neat PTQ10 film (**f**), neat IDIC film (**g**), and PTQ10: IDIC blend films without (as-cast) (**h**), with TA treatment (**i**), and with TA + SA treatment (**j**)

Morphology of the active layer is a critical factor to determine the photovoltaic performance of the PSCs^42,43^. Here, the grazing incident wide-angle X-ray diffraction (GIWAXS) was employed to study the effect of different post treatments on the molecular packing and material crystallinity features within the PTQ10: IDIC blend films. Figure 3 shows the plots and images of GIWAXS measurements. For the neat PTQ10 film, the laminar diffraction peaks and π–π stacking diffraction peaks located at 0.28 Å^−1^ and 1.76 Å^−1^ (Fig. 3a, f) respectively, corresponding to the lamellar distance of 22.44 Å and π–π stacking distance of 3.57 Å, and the neat IDIC film shows the lamellar distance of 15.71 Å and π–π stacking distance of 3.50 Å (Fig. 3b, g). The strong π–π stacking diffraction peaks in the out-of-plane (OOP) direction and weak π–π stacking diffraction peaks in the in-plane (IP) direction of both neat PTQ10 and IDIC film suggest strong preference of face-on orientation in the vertical direction of substrate for the molecular packing, which is beneficial for efficient charge transport. The GIWAXS plots of blend films demonstrate microstructural features of its individual components. For the PTQ10: IDIC blend films with TA treatment (Fig. 3d, i), the molecular packing exhibit preferred and enhanced face-on orientation at 1.81 Å^−1^ with stronger and sharper π–π stacking peaks in OOP direction in comparison with the as-cast films. Moreover, the π–π stacking diffraction peaks intensity at 1.81 Å^−1^ in OOP direction was further improved with TA + SA treatment (Fig. 3e, j), indicating the enhanced charge transport behavior in the vertical direction of substrate in the devices treated by TA or TA + SA. Furthermore, the π–π stacking distance is decreased to 3.47 Å for the TA-treated and TA + SA-treated blend films in comparison with the as-cast films with the π–π stacking distance of 3.53 Å, suggesting more tighter molecular packing after the treatment. The results indicate that the preferred face-on orientation, the closer π–π stacking, and the higher crystalline characteristics of the post-treated blend films (especially for the TA + SA-treated blend films), assisted charge transport, suppressed charge carrier recombination, and eventually improved the photovoltaic performance. Furthermore, transmission electron microscope (TEM) measurements were carried out to study the effect of processing conditions on the morphology. From the TEM images (Supplementary Fig. 7), the PTQ10: IDIC blend films show obviously fibrillary networks and increased domain size after TA or TA + SA treatments.

**Fig. 4:**
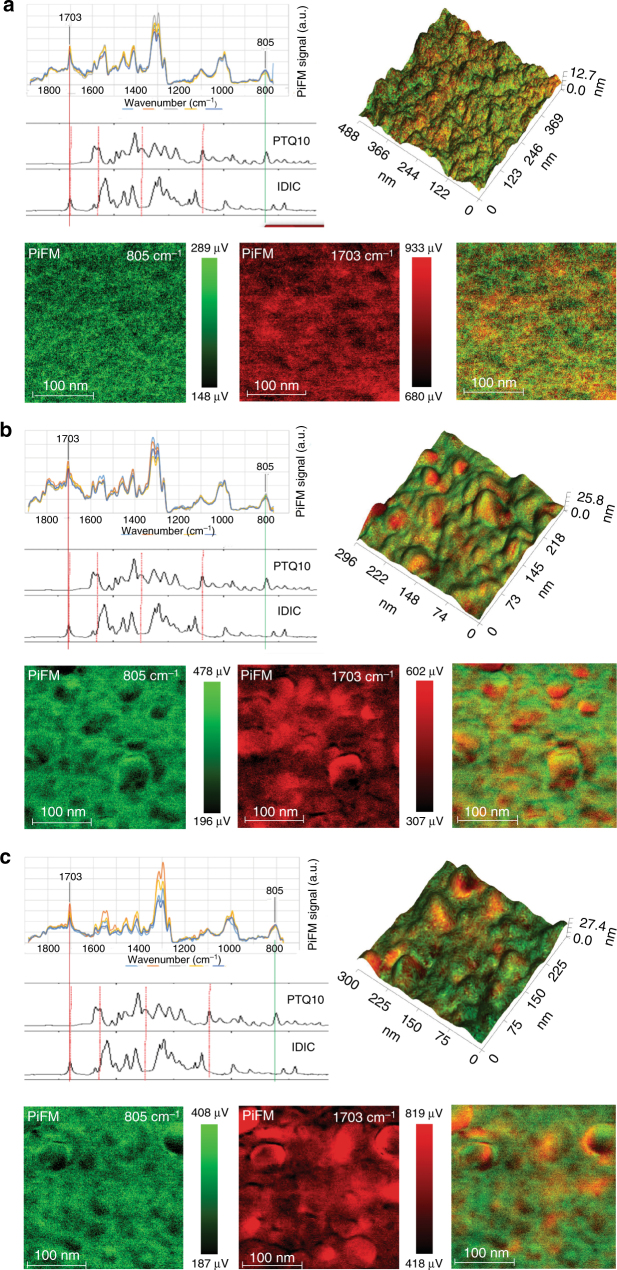
FTIR spectra and PiFM topography images. FTIR spectra and PiFM images of PTQ10: IDIC blend films based on FTIR absorption at different wave numbers (PTQ10, 805 cm^−1^ and IDIC, 1703 cm^−1^): without (as-cast) (**a**), with TA treatment (**b**), and with TA + SA treatment (**c**)

In addition, photoinduced force microscopy (PiFM)^44^, an emergent technology that demonstrates the spatially nm-scale patterns of the individual chemical components in their blend films^45^, was used to study the effect of processing conditions on the morphology. The PiFM images at the characteristic Fourier transform infrared (FTIR) wavelengths corresponding to absorption peaks of polymer donor PTQ10 (805 cm^−1^) and acceptor IDIC (1703 cm^−1^) with different post treatments are shown in Fig. 4. From the PiFM images, the TA-treated PTQ10: IDIC blend films (Fig. 4b) show obviously increased phase separation and domain size in comparison with the as-cast films (Fig. 4a), and the further increased phase separation and domain size was observed in the TA + SA-treated films (Fig. 4c). The PiFM results are consistent with the GIWAX and TEM measurements. The results indicate that the gradually enhanced photovoltaic properties of the devices with TA and TA + SA treatment could be ascribed to the larger phase domains and the more continuous donor/acceptor nano-scale phase-separated interpenetrating networks.

### Thickness dependence of the photovoltaic performance

**Fig. 5:**
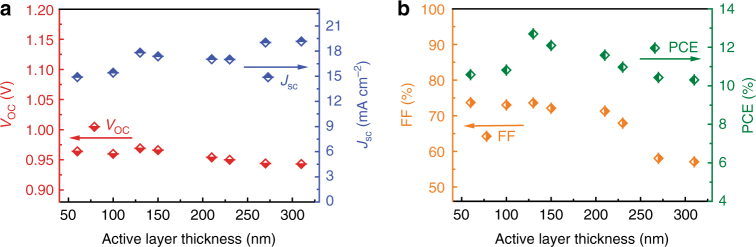
Thickness dependence of the photovoltaic performance. Plots of *V*_oc_ or *J*_sc_ (**a**) and FF or PCE (**b**) vs. the active layer thickness ranging from 60 to 310 nm for the traditional structured PSCs

For large area fabrication of the PSCs, the active layer thickness is difficult to be precisely controlled^46^. Therefore, it is crucial to develop the thickness-insensitive polymer donors with excellent photovoltaic performance. Here, we investigated the effect of active layer thickness on the photovoltaic performance of the traditional structured PSCs based on PTQ10: IDIC with active layer thickness ranging from 60 to 310 nm. Figure 5a, b shows the thickness dependence of photovoltaic performance, and Supplementary Table 2 lists the corresponding photovoltaic parameters of the devices. The *V*_oc_ values are nearly constant with a slightly decrease for the active layers thicker than 130 nm (Fig. 5a). The *J*_sc_ values show an increasing trend from approximately 15 to 19 mA cm^−2^ with the increase of the active layer thickness. The changes of *J*_sc_ should be the trade-off results between absorbance and charge recombination, the thicker PTQ10: IDIC active layers will enhance the light harvest which is beneficial to higher *J*_sc_, but it also increases charge recombination which will decrease *J*_sc_. FF shows relatively significant thickness-dependent behavior, the FF values remain high and close to 72% even with the active layer thickness of up to 210 nm, but it sharply decreased to ca. 58% as the active layer thickness increased to 310 nm (Fig. 5b), which could be due to the increased series resistance of the PSCs with the too thick active layer. As a result, the highest PCE of 12.70% is obtained for the device with the active layer thickness of 130 nm. It should be noted that the high PCE of 11.59% was also obtained even the active layer thickness increased to 210 nm. Amazingly, even for the PSCs with a thicker active layer of 310 nm, its PCE still reached a high value of 10.31%. The excellent and thickness-insensitive photovoltaic performance of the PSCs based on PTQ10: IDIC makes it a strong candidate for large area fabrication and commercial applications of the PSCs.

### Cost and PCE analysis

PTQ10 has extremely simple molecular structure (Fig. 1a) in comparison with the efficient polymer donors reported in literatures, and it can be easily synthesized via only two-step reactions from initial raw materials and once purification. Besides, all of the raw materials, such as 3,6-dibromo-4,5-difluorobenzene-1,2-diamine, glyoxylic acid, 1-bromo-2-hexyldecane, and 2,5-bis(trimethylstannyl)thiophene, are inexpensive and available from bulk chemical suppliers. Consequently, PTQ10 exhibits extremely high synthetic accessibility and low cost for commercial production, potentially reducing the energy pay back times.

**Fig. 6:**
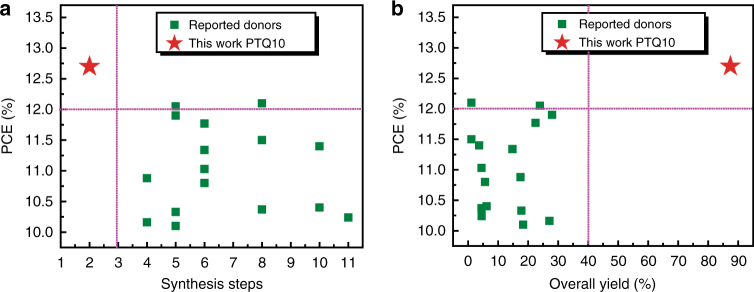
Cost and PCE analysis of the PSCs. Plots of PCE vs. synthesis steps (**a**) and overall yield (**b**) of the polymer donors reported in literatures with PCE over 10%

Figure 6a, b displays the plots of PCE values versus synthesis steps and overall yield of PTQ10 respectively in comparison with the efficient polymer donors with PCE over 10% reported in literatures. The corresponding statistical photovoltaic parameters are listed in Supplementary Table 3. It can be seen from the figures that the PTQ10-based PSCs has the highest PCE (12.70%) with the minimum synthesis steps of 2 steps (which is ca. one-third or one-fifth of that for the other efficient polymer donors) and the highest overall yield of 87.4% (which is ca. 5–20 times of that for the other efficient polymer donors). The less synthetic steps of PTQ10 should be ascribed to its simplest D–A structure, and the high overall yield is benefitted from the high yield of its two stepwise reactions (91% and 96%) and only once purification. The ultimate cost of organic photovoltaic materials reduces linearly with the reduction of the number of synthetic steps^30^, thus the ultimate cost of PTQ10 is only few tenths of other efficient donors. Besides, the high overall yield further increases its low-cost advantages. Obviously, PTQ10 possesses great superiority in both cost and photovoltaic performance, which will lead to a bright future for the commercial application of PSCs.

## Discussion

In conclusion, a low-cost polymer donor PTQ10 with only two synthetic steps and high yield of 87.4% was designed and synthesized in pursuing low-cost polymer donor materials for future application of PSCs. PTQ10 possesses a broad and strong absorption band in the wavelength range of 450–620 nm with a medium bandgap of 1.92 eV and lower lying HOMO energy level of −5.54 eV. The traditional structured PSCs using PTQ10 as donor and a narrow bandgap *n*-OS IDIC as acceptor demonstrated a high PCE of 12.70%, and its photovoltaic performance exhibits insensitivity of active layer thickness between 100 and 300 nm, which is conductive to the large area fabrication of PSCs. The PCE of the inverted structured PSCs based on PTQ10: IDIC also reached 12.13% which was confirmed to be 12.0% by NIM. In comparison with the polymer donors reported in literatures with PCE over 10%, PTQ10 shows the great advantages of low cost (benefiting by less synthetic steps and high overall yield) and high photovoltaic performance. Therefore, we believe that PTQ10 is a highly promising polymer donor for large area fabrication of PSCs, and it will push the commercial application of PSCs forward.

## Methods

### Materials and synthesis

IDIC was purchased from Solarmer Materials Inc. Other chemicals and solvents were obtained from J&K, Alfa Aesar, and TCI Chemical Co., respectively. The monomer compound 2 and polymer PTQ10 was synthesized according to the synthetic route shown in Fig. 1c. The detailed synthesis procedures are described in the following^47^.

5,8-dibromo-6,7-difluoro-2-(2-hexyldecyloxy)quinoxaline (compound 2):

To a two-necked, round-bottom flask, 3,6-dibromo-4,5-difluorobenzene-1,2-diamine (compound 1) (906 mg, 3 mmol), glyoxylic acid (222 mg, 3 mmol), and acetic acid (30 mL) are added. The mixture is warmed to 40 °C for 10 min, and then the solution is stirred at room temperature for 3 h. The precipitate is collected by filtration and dried to get a white solid without further purification. The white solid (1 g, 2.94 mmol), potassium tert-butanolate (395 mg, 3.53 mmol), and 1-bromo-2-hexyldecane (897 mg, 2.94 mmol) are dissolved in methanol (30 mL). The mixture is refluxed for 12 h, then cooled to room temperature. After that, the reaction mixture is poured into saturated NH_4_Cl solution, extracted with dichloromethane, and washed with water. The organic extraction is dried over anhydrous MgSO_4_, and the solvent is evaporated under reduced pressure. Compound 2 is obtained as colorless oil (1.54 g, 2.73 mmol) from the product through column chromatography on silica gel, with an overall yield of 91%. ^1^H NMR (400 MHz, CDCl_3_): *δ* (p.p.m.) 8.50 (s, 1H), 4.49 (d, *J* = 5.7 Hz, 2H), 1.95–1.86 (m, 1H), 1.55–1.36 (m, 8H), 1.35–1.18 (m, 16H), 0.92–0.83 (m, 6H). ^13^C NMR (100 MHz, CDCl_3_): *δ* (p.p.m.) 158.69, 151.82, 149.33, 146.88, 140.66, 136.31, 133.26, 109.75, 107.60, 70.50, 37.46, 31.87, 31.36, 29.98, 29.61, 29.31, 26.84, 22.67, 14.09.

Poly[(thiophene)-alt-(6,7-difluoro-2-(2-hexyldecyloxy)quinoxaline] (PTQ10):

The polymer PTQ10 is synthesized according to still-coupling poly-condensation between compound 2 and 2,5-bis(trimethylstannyl)thiophene under protection of argon. Compound 2 (112.8 mg, 0.2 mmol), 2,5-bis(trimethylstannyl)thiophene (82 mg, 0.2 mmol), and anhydrous toluene (10 mL) are added to a 25-mL double-neck round-bottom flask. The flask is flushed with argon for 10 min, and then tetrakis(triphenylphosphine)palladium(0) (Pd(PPh_3_)_4_, 8 mg) is added. After another flushing with argon for 15 min, the reactant is heated to reflux for 32 h. Then the reactant is cooled down to room temperature, and extracted by Soxhlet extractor with methanol, hexane, and chloroform one by one. The polymer (93 mg, yield 96%) is recovered from the chloroform extract by precipitation in methanol and dried under vacuum. GPC: *M*n = 39.1 kDa; *M*w/*M*n = 2.1. Anal. Calcd for C_28_H_36_F_2_N_2_OS (%): C, 69.10; H, 7.46; N, 5.76. Found (%): C, 68.08; H, 7.48; N, 5.71. ^1^H NMR (CDCl_3_, 400 MHz): *δ* (p.p.m.) 8.81–7.72 (br, 3H), 4.89–4.03 (br, 2H), 2.43–0.53 (br, 31H).

### General characterization

^1^H NMR and ^13^C NMR spectra of the corresponding compounds were measured on a Bruker DMX-400 spectrometer using *d*–chloroform as solvent and trimethylsilane as the internal reference. High-temperature gel permeation chromatography (GPC) measurements were carried out on Agilent PL-GPC 220 instrument, using 1,2,4-trichlorobenzene as the eluent at 160 °C. Thermogravimetric analysis (TGA) was measured on a Perkin-Elmer TGA-7 thermogravimetric analyzer with a heating rate of 10 °C min^−1^ under a nitrogen flow rate of 100 mL min^−1^. UV–visible absorption spectra were measured on a Hitachi U-3010 UV–vis spectrophotometer. Electrochemical cyclic voltammograms was measured on a Zahner IM6e Electrochemical Workstation under a nitrogen atmosphere, with a Pt disk as working electrode, a Ag/AgCl as reference electrode, and a Pt wire as counter electrode in acetonitrile solution of tetrabutylammonium hexafluorophosphate (*n*-Bu_4_NPF_6_), and ferrocene/ferrocenium (Fc/Fc^+^) couple was used as an internal reference.

### Mobility measurements

Hole mobility and electron mobility were measured using the space charge limited current (SCLC) method. The hole-only device with the device structure of ITO /PEDOT: PSS/PTQ10: IDIC/Au was used to measure the hole mobility, and the electron-only device with the device structure of ITO/ZnO/PTQ10: IDIC/PDINO Al was used to measure the electron mobility. The hole and electron mobilities were calculated by MOTT–Gurney equation:

1 $$J = \frac{{9\varepsilon _{\mathrm{0}}\varepsilon _{\mathrm{r}}\mu V^2}}{{8L^3}},$$

where *J* is the current density, *ε*_0_ the dielectric constant of empty space, *ε*_r_ the relative dielectric constant of active layer materials which is taken to be 3 in the calculation, *μ* the charge mobility, *V* the internal voltage in the device, and *V* = *V*_appl_−*V*_bi_−*V*_s_, where *V*_appl_ is the voltage applied to the devices, *V*_bi_ the built-in voltage from the relative work function difference between the two electrodes (0.2 V for the hole-only device and 0 V for the electron-only device), *V*_s_ the voltage drop from the series resistance, and *L* the thickness of the active layers.

### GIWAXS measurements

GIWAXS measurements were carried out using small angle X-ray scattering system (XEUSS, FRANCE Xenocs SA). The samples for the GIWAXS measurements were prepared on Si substrates using chloroform solutions of the samples. The 10 keV X-ray beam was incident at a grazing angle of 0.13–0.17°. The scattered X-rays were detected using a Dectris Pilatus 2M photon counting detector.

### TEM characterization

The TEM images were obtained on JEM-1011. The active layer films for the TEM measurements were spin-coated onto ITO/PEDOT: PSS substrates, and the substrates with the active layers were submerged in deionized water to make the active layers fall off, then the active layer films were picked up by copper grids for TEM measurements.

### Device fabrication and characterization

The PSCs based on PTQ10: IDIC were fabricated with a device structure of ITO/PEDOT: PSS/PTQ10: IDIC/PDINO/Al. A thin layer of PEDOT: PSS was prepared on precleaned ITO glass through spin-coating a PEDOT: PSS aqueous solution (Baytron P VP AI 4083 from H. C. Starck) at 2000 rpm and dried subsequently at 150 °C for 15 min in air. Then the device was transferred to a glove box filled with nitrogen, in which the active layer of PTQ10: IDIC was spin-coated from its chloroform solution onto the PEDOT: PSS layer at 3500 rpm. After spin-coating, the active layers were annealed at 140 °C for 5 min for the devices with TA treatment, and then the active layers were treated by chloroform solvent for 30 s for the devices with solvent annealing treatment. The thickness of the active layer is ca. 130 nm. Then methanol solution of PDINO at a concentration of 1.0 mg mL^−1^ was deposited upon the active layer at 3000 rpm to afford a PDINO cathode buffer layer with thickness of ca. 10 nm. Finally, cathode metal Al was deposited onto the cathode buffer layer PDINO at a pressure of ca. 5.0 × 10^−5^ Pa. The effective area of the devices was 4.7 mm^2^ which was defined by Optical microscope (Olympus BX51). The current density–voltage (*J–V*) curves of the PSCs were measured by scanning voltage from −1.5 V to 1.5 V with a voltage step of 10 mV and delay time of 1 ms on Keithley 2450 Source-Measure Unit in a glove box filled with nitrogen (oxygen and water contents are smaller than 0.1 ppm). Oriel Sol3A Class AAA Solar Simulator (model, Newport 94023A) with a 450 W xenon lamp and an air mass (AM) 1.5 filter was used as the light source. The light intensity was calibrated to be 100 mW cm^−2^ by a Newport Oriel 91150V reference cell. For accurately measuring the photocurrent, mask with an area of 2.2 mm^2^ was used to define the effective area of the devices. The results with or without mask showed consistent values with relative errors within 0.5% (the devices with mask give slightly higher PCE due to its slightly higher FF). The PCE results in the manuscript are obtained from the measurement without mask and PCE statistics were obtained using more than 20 individual devices fabricated under the same conditions. The EQE was measured by Solar Cell Spectral Response Measurement System QE-R3-011 (Enli Technology Co., Ltd., Taiwan). The light intensity at each wavelength was calibrated with a standard single-crystal Si photovoltaic cell.

Inverted devices were fabricated with a structure of ITO/ZnO/PTQ10: IDIC /MoO_3_/Ag. The ZnO precursor solution was prepared by dissolving 0.14 g of zinc acetate dehydrate (Zn(CH_3_COO)_2_⋅2H_2_O, 99.9%, Aldrich) and 0.5 g of ethanolamine (NH_2_CH_2_CH_2_OH, 99.5%, Aldrich) in 5 ml of 2-methoxyethanol (CH_3_OCH_2_CH_2_OH, 99.8%, J&K Scientific). A thin layer of ZnO was deposited through spin-coating the ZnO precursor solution on precleaned ITO glass at 5000 rpm and baked subsequently at 200 °C for 30 min. Then the device was transferred into a glove box filled with nitrogen, in which the active layer of PTQ10: IDIC (1: 1.5, w/w) was spin-coated from its chloroform solution onto the ZnO at 3500 rpm. After that, the active layers were annealed at 140 °C for 5 min for the devices with TA treatment, and treated by chloroform solvent for 30 s for the devices with solvent annealing treatment. The thickness of the active layer is ca. 130 nm. Finally, a layer of ca. 5 nm MoO_3_ and then a Ag layer of ca. 160 nm were evaporated subsequently under high vacuum.

### Device stability measurements

The inverted structured PSCs with device structure of ITO/ZnO/PTQ10: IDIC /MoO_3_/Ag for stability measurements were encapsulated by ultraviolet-curable epoxy and thin glass slides and stored in nitrogen and air atmosphere, respectively.

### Data availability

The data that support the findings of this study are available from the corresponding author upon reasonable request.

## Electronic supplementary material

Supplementary Information
