# Supplementary material for: A low cost and high performance polymer donor material for polymer solar cells
Source: Nat Commun. 2018 Feb 21;9:743. doi: 10.1038/s41467-018-03207-x (PMC5821836; doi:10.1038/s41467-018-03207-x)
Supplement: Supplementary file 1 — Supplementary Information [file 41467_2018_3207_MOESM1_ESM.pdf]

Supplementary Figures

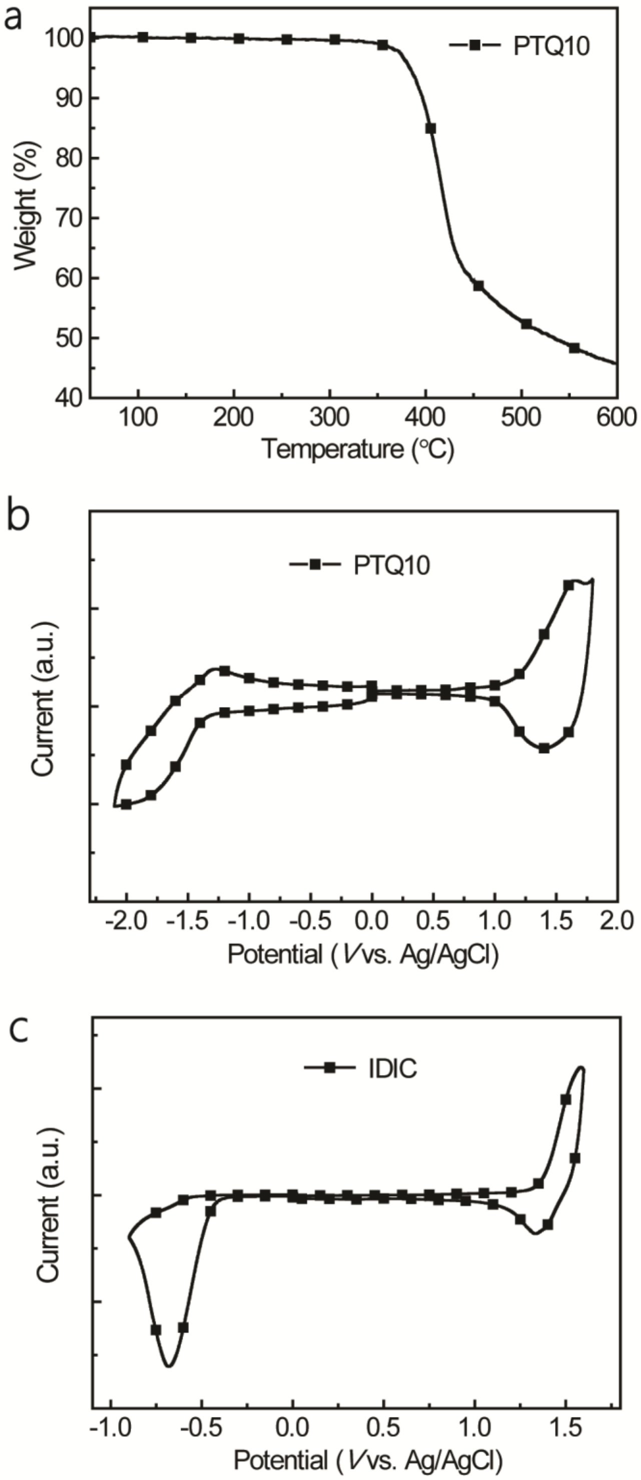

Supplementary Figure 1 │ TGA plot and cyclic voltammograms of PTQ10 and IDIC. a, TGA plot of the polymer PTQ10. Cyclic voltammograms of the polymer (**b**) PTQ10 and (**c**) IDIC films on a platinum electrode measured in 0.1 mol L^{-1} Bu_4NPF_6 acetonitrile solution at a scan rate of 20 mV s^{-1}.

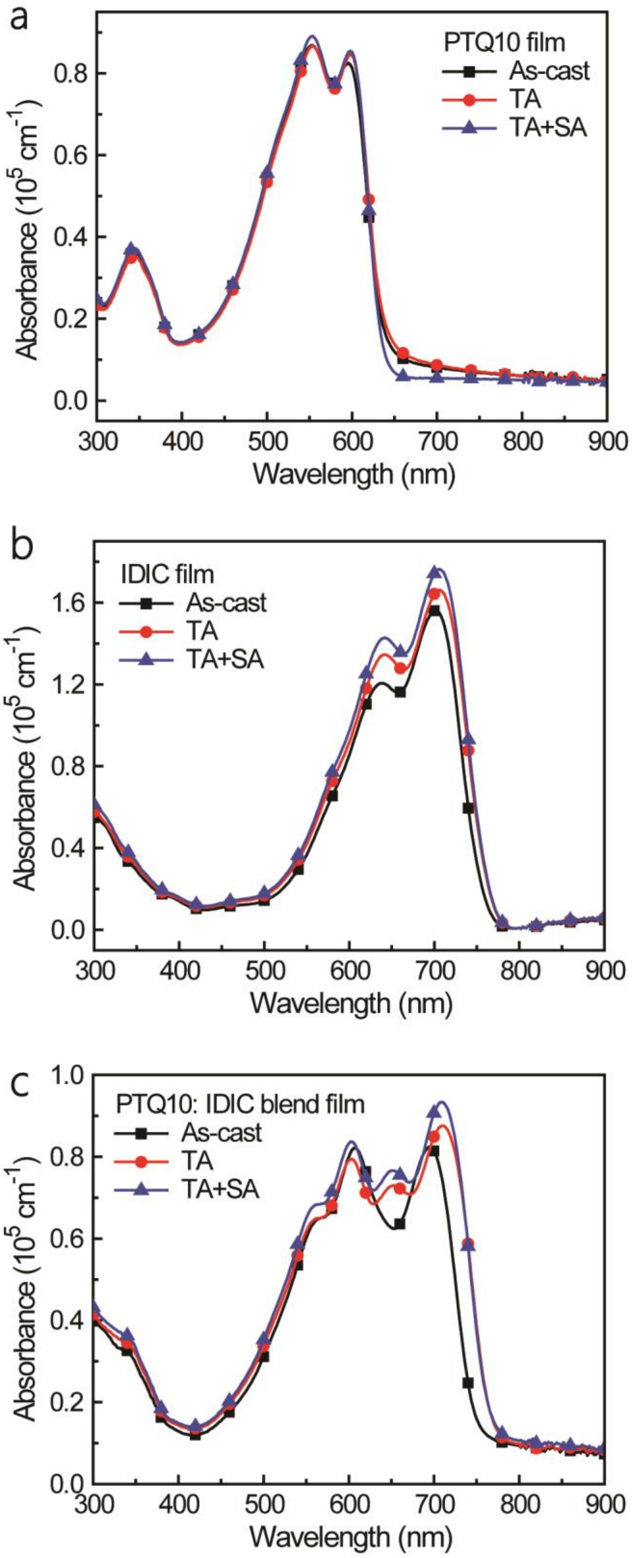

Supplementary Figure 2 | Absorption spectra of PTQ10: IDIC films. Absorption spectra of (**a**) PTQ10, (**b**) IDIC and (**c**) PTQ10: IDIC films without (as-cast) and with TA or TA+SA treatments.

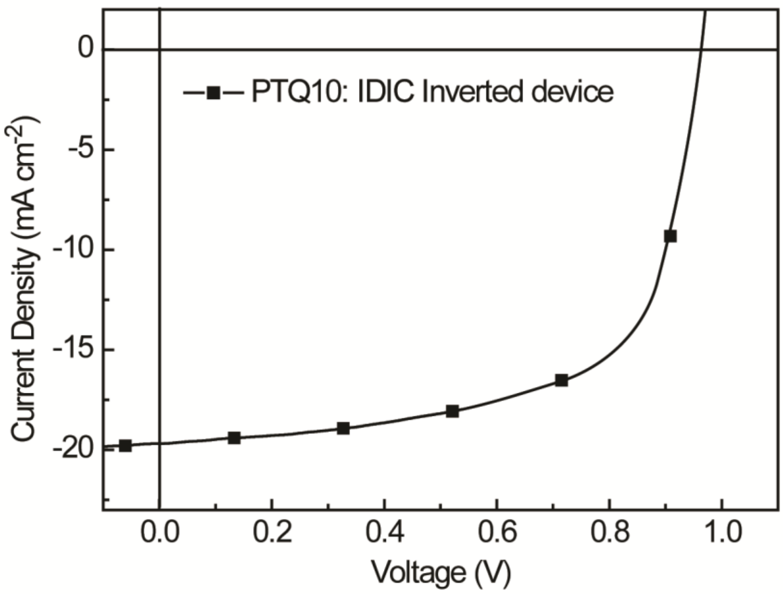

Supplementary Figure 3 | Photovoltaic performance of the inverted structured PSCs. *J-V* curve of the inverted structured PSCs based on PTQ10: IDIC (1:1.5, w/w) with thermal annealing at 140°C for 5 min and solvent vapor annealing by chloroform solvent for 30 s, under the illumination of AM1.5G, 100 mW cm^{-2}.

中 国 计 量 科 学 研 究 院

证书编号 GXtc2017-1677
Certificate No.

测 试 结 果
Calibration Results

1. 测试条件 Test Conditions:

标准太阳电池：单晶硅（81#）；
Reference Solar Cell: mono-Si;
标准太阳电池的标定值：125.68 mA；
CV of Reference Solar Cel: 125.68 mA;
太阳模拟器等级：AAA 级（双光源）；
Solar Simulator Classification: AAA (double-light source);
温度传感器/控制系统：无；
Teperature Sensor/Control System: None;
扫描方向：反扫。
Scan Direction: reverse.

2. I-V 特性参数 I-V Characteristic parameters:

以上述标准太阳电池标定太阳模拟器辐照度至 1000 W/m^2，校准被测太阳电池的 I-V 特性曲线和参数如下：

By using the above reference solar cell to calibrate the solar simulator's irradiance to 1000 W/m^2, the I-V characterisitc curve and parameters as follows:

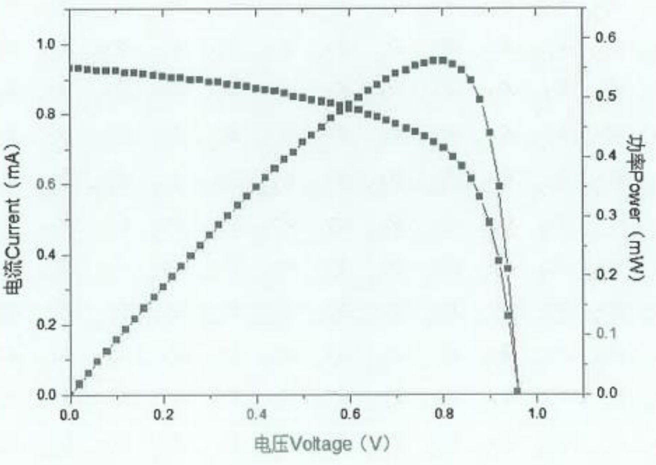

第 3 页 共 4 页

Supplementary Figure 4 | Test report of the PCE confirmation of the PSCs. Test report (the third page) of the PSCs based on PTQ10: IDIC with an inverted device structure of ITO /ZnO /PTQ10: IDIC /MoO_3 /Ag at the optimized device fabrication

conditions of donor: acceptor weight ratio of 1:1.5, active layer thickness of 130 nm with thermal annealing at 140°C for 5 min and solvent vapor annealing by chloroform solvent for 30 s. (from The National Institute of Metrology (NIM) of China) (all the four pages of the report is available from the first and corresponding authors)

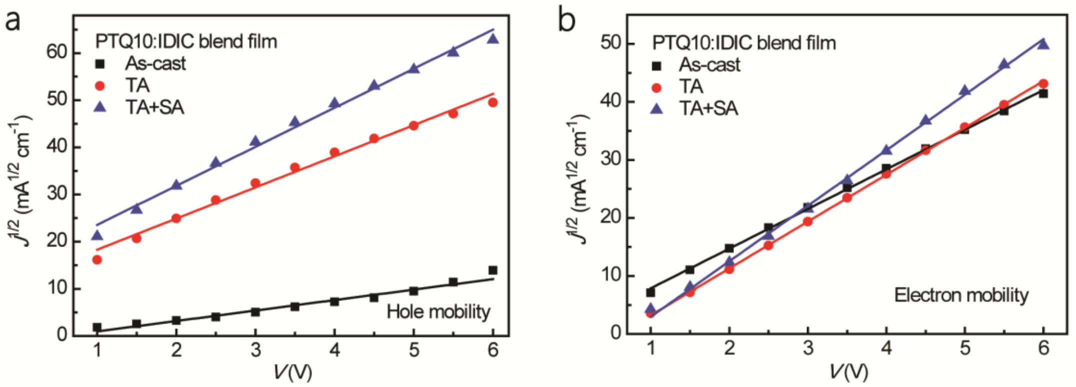

Supplementary Figure 5 | $J^{1/2}$~V ($V = V_{appl}$-V_{bi}-V_s) characteristics for the PSCs. a, Hole-only devices (ITO /PEDOT: PSS /PTQ10: IDIC /Au) and (**b**) electron-only devices (ITO /ZnO /PTQ10: IDIC /PDINO /Al).

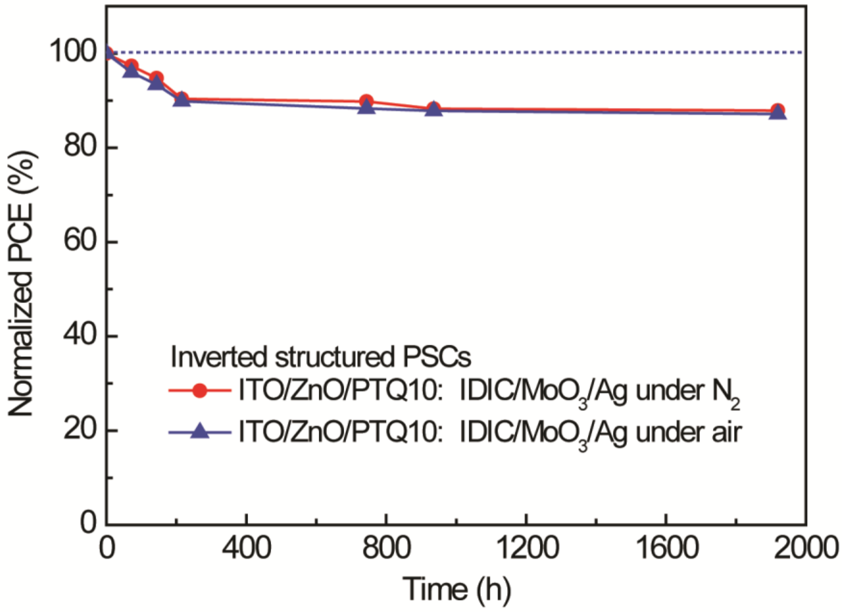

Supplementary Figure 6 | Device stability of the inverted structured PSCs. Plots of normalized PCE against time of the inverted structured PSCs with devices structure of ITO /ZnO /PTQ10: IDIC /MoO_3 /Ag with simple encapsulation and storage in nitrogen and air atmosphere respectively.

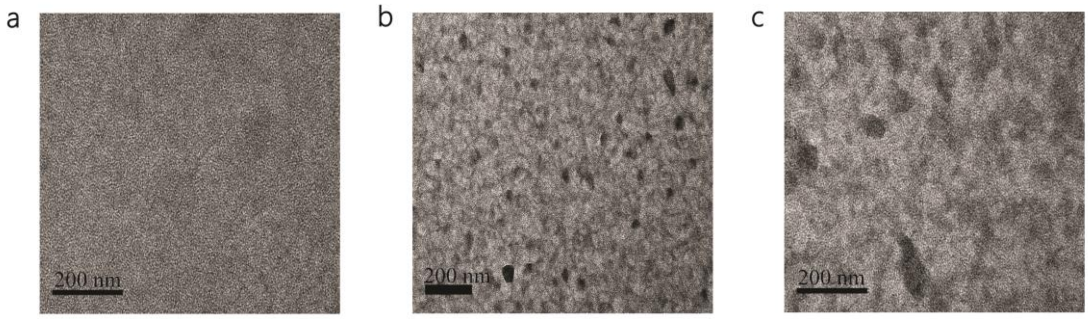

Supplementary Figure 7 | TEM images of PTQ10: IDIC blend films. TEM images of PTQ10: IDIC blend films (**a**) without (as-cast), (**b**) with TA treatment and (**c**) with TA+SA treatment.

Supplementary Tables

Supplementary Table 1│ Photovoltaic parameters of the PSCs based on PTQ10: IDIC with the polymer donor PTQ10 from five batches

Batch number	M_n [kDa]	PDI	V_{oc} [V]	J_{sc} [mA cm^{-2}]	FF [%]	PCE [%]
1	39.1	2.1	0.969	17.81	73.60	12.70
2	36.9	2.3	0.966	17.32	73.87	12.36
3	33.7	2.1	0.971	17.38	74.11	12.51
4	45.2	2.5	0.962	16.87	73.33	11.90
5	17.8	2.0	0.970	16.89	74.10	12.15

Supplementary Table 2│ Effect of the active layer thickness on the photovoltaic performance of the PSCs based on PTQ10: IDIC

Thickness [nm]	V_{oc} [V]	J_{sc} [mA cm^{-2}]	FF [%]	PCE [%]
60	0.964	14.90	73.70	10.58
100	0.960	15.43	73.03	10.82
130	0.969	17.81	73.60	12.70
150	0.966	17.37	72.11	12.10
210	0.954	17.03	71.30	11.59
230	0.950	17.00	67.94	10.97
270	0.944	19.03	58.06	10.43
310	0.943	19.16	57.10	10.31

Supplementary Table 3│ PCE, synthesis steps and overall yield of the various donors reported in literatures with PCE over 10%

**Donor**: Acceptor	**Synthesis steps**	**Overall yield [%]**	**PCE [%]**	**References**
**PB3T**: IT-M	5	28.01	11.90	1
**PDCBT**: ITIC	4	27.13	10.16	2
**PBQ-4F**: ITIC	6	14.82	11.34	3
**PBDB-T**: IT-M	5	23.94	12.05	4
**PTFBDT-BZS**: IDIC	6	4.48	11.03	5
**PDBT-T1**: IDIC	8	4.5	10.37	5
**PTFB-O**: ITIC-Th	4	17.53	10.88	6
**FTAZ**: INIC3	8	1.1	11.50	7
**J61**: m-ITIC	6	22.51	11.77	8
**J71**: ITIC	10	3.7	11.40	9
**PNTz4T**: $PC_{71}BM$	5	18.4	10.10	10
**PTzBI**: ITIC	11	4.55	10.24	11
**FTAZ**: ITIC-Th1	8	1.1	12.10	12
**PBTff4T-2OD**: $PC_{71}BM$	10	6.2	10.40	13
**PffBT4T-2OD**: $TC_{71}BM$	6	5.7	10.80	13
**NT812**: $PC_{71}BM$	5	17.8	10.33	14
**PTQ10**: IDIC	2	87.4	12.70	This work

Supplementary Notes

Supplementary Note 1. The calculation of electronic energy levels of donor PTQ10 and acceptor IDIC

The HOMO/LUMO energy levels (E_{HOMO}/E_{LUMO}) can be calculated from onset oxidation and reduction potentials (φ_{ox}/φ_{red}) according to the equation of E_{HOMO}/E_{LUMO}= -e (φ_{ox}/φ_{red} – φ_{Fc/Fc^+} + 4.8) eV, where φ_{Fc/Fc^+} was measured to be 0.44 V *vs.* Ag/AgCl. From cyclic voltammogram of PTQ10, the φ_{ox}/φ_{red} of are 1.18 V and -1.38 V *vs*. Ag/AgCl (Supplementary Fig. 1b). So the E_{HOMO} and E_{LUMO} of PTQ10 were calculated to be -5.54 eV and -2.98 eV respectively. From cyclic voltammogram of IDIC, the φ_{ox}/φ_{red} of are 1.38 V and -0.46 V *vs*. Ag/AgCl (Supplementary Fig. 1c). So the E_{HOMO} and E_{LUMO} of IDIC were calculated to be -5.74 eV and -3.90 eV respectively.

Supplementary Note 2. The statement of the statistical results in Supplementary Table 3

As the starting materials and monomers (such as organotin compounds and acceptor units) of partial polymer donors are purchased in high price and small amount from companies (In fact, those purchased materials can only meet the needs of research, not for large-scale commercialization), the statistical results in Supplementary Table 3 are qualitative rather than quantitative. Despite this, it is un-deniable that the polymer donor PTQ10 has great superiority in both cost and photovoltaic performance in comparison with other high performance polymer donors.

Supplementary References

1. Liu, D. *et al*. Molecular design of a wide-band-gap conjugated polymer for efficient fullerene-free polymer solar cells. *Energy Environ. Sci.* **10**, 546-551 (2017).
2. Qin, Y. *et al*. Highly efficient fullerene-free polymer solar cells fabricated with polythiophene derivative. *Adv. Mater.* **28**, 9416-9422 (2016).
3. Zheng, Z. *et al*. Efficient charge transfer and fine-tuned energy level alignment in a THF-processed fullerene-free organic solar cell with 11.3% efficiency. *Adv. Mater.* **29**, (2017).
4. Li, S. *et al*. Energy-level modulation of small-molecule electron acceptors to achieve over 12% efficiency in polymer solar cells. *Adv. Mater.* **28**, 9423-9429 (2016).
5. Lin, Y. *et al*. Mapping polymer donors toward high-efficiency fullerene free organic solar cells. *Adv. Mater.* **29**, 1604155 (2017).
6. Li, Z. *et al*. Donor polymer design enables efficient non-fullerene organic solar cells. *Nat. Commun.* **7**, 13094 (2016).
7. Dai, S. *et al*. Fused nonacyclic electron acceptors for efficient polymer solar cells. *J. Am. Chem. Soc.* **139**, 1336-1343 (2017).
8. Yang, Y. *et al*. Side-chain isomerization on an n-type organic semiconductor ITIC acceptor makes 11.77% high efficiency polymer solar cells. *J. Am. Chem. Soc.* **138**, 15011-15018 (2016).
9. Bin, H. *et al*. 11.4% Efficiency non-fullerene polymer solar cells with trialkylsilyl substituted 2D-conjugated polymer as donor. *Nat. Commun.* **7**, 13651 (2016).
10. Vohra, V. *et al*. Efficient inverted polymer solar cells employing favourable molecular orientation. *Nat. Photon.* **9**, 403-408 (2015).
11. Fan, B. *et al*. High-performance nonfullerene polymer solar cells based on imide-functionalized wide-bandgap polymers. *Adv. Mater.* 2017.
12. Zhao, F. *et al*. Single-junction binary-blend nonfullerene polymer solar cells with 12.1% efficiency. *Adv. Mater.* 2017.
13. Liu, Y. *et al*. Aggregation and morphology control enables multiple cases of high-efficiency polymer solar cells. *Nat. Commun.* **5**, 5293 (2014).
14. Jin, Y. *et al.* A novel naphtho[1,2-c:5,6-c']bis([1,2,5]thiadiazole)-based narrow-bandgap pi-conjugated polymer with power conversion efficiency over 10%. *Adv. Mater.* **28**, 9811-9818 (2016).
